# *Cathepsin L* Contributes to Reproductive Diapause by Regulating Lipid Storage and Survival of *Coccinella septempunctata* (Linnaeus)

**DOI:** 10.3390/ijms24010611

**Published:** 2022-12-29

**Authors:** Junjie Chen, Penghui Guo, Yuyan Li, Weiwei He, Wanbin Chen, Zhongjian Shen, Maosen Zhang, Jianjun Mao, Lisheng Zhang

**Affiliations:** Key Laboratory of Natural Enemy Insects, Ministry of Agriculture and Rural Affairs, Institute of Plant Protection, Chinese Academy of Agricultural Sciences, No. 2, West Yuan Ming Yuan Road, Beijing 100193, China

**Keywords:** *Coccinella septempunctata*, *cathepsin L*, diapause, survival rate, lipid accumulation

## Abstract

Cathepsin L protease, which belongs to the papain-like cysteine proteases family, is an important player in many physiological and pathological processes. However, little was known about the role of *cathepsin L* in ladybird beetles (*Coccinella septempuctata* Linnaeus) during diapause. Here, we analyzed the characteristics of *cathepsin L* (*CsCatL*) in the females of *C. septempunctata* and its role during the diapause of the ladybeetle. *CsCatL* was cloned and identified from beetle specimens by rapid amplification of cDNA-ends (RACE). The cDNA sequence of *CsCatL* was 971 bp in length, including an 843 bp open reading frame encoding a protein of 280 amino acids. It was identified as the *cathepsin L* group by phylogenetic analysis. Knockdown of *CsCatL* by RNA interference led to decreased expression levels of fatty acid synthase 2 (fas 2) genes and suppressed lipid accumulation. Furthermore, silencing the *CsCatL* gene distinctly reduced diapause-related features and the survival of female *C. spetempunctata* under diapause-inducing conditions. The results suggested that the *CsCatL* gene was involved in fatty acid biosynthesis and played a crucial role in the survival of adult *C. septempunctata* during the diapause preparation stage.

## 1. Introduction

Diapause is a physiological and ecological adaptive strategy for coping with adverse environmental conditions, such as low temperature, desiccation, and absence of food [1]. It is essential to the survival of individuals and is accompanied by developmental arrest, accumulation of energy reserves, metabolic decline, increased stress-resistance, and extended lifespan [2,3]. The development of insect diapause is a complex and dynamic process that can be distinguished into three main major phases: pre-diapause, diapause and post-diapause [4]. To successfully enter diapause, insect individuals usually undergo different behavioral and physiological changes, including the accumulation of energy reserves, migration, or location of suitable micro-habitats during the preparation phase [5].

In many insects, lipid accumulation is an obvious phenotype observed during the pre-diapause stage (*Colaphellus bowringi*, *Culex pipiens* and *Coccinella septempunctata*) [6,7,8,9,10]. Lipid reserves are the most important resources for insects to meet energy demand during the dormancy state known as diapause [11]. Insects accumulate massive lipids prior to diapause and a failure to accumulate adequate amounts of lipids leads to incomplete diapause and possibly death in prolonged period of diapause with no or reduced feeding [7,8,9,12].

Natural predators are important for pest control in agricultural systems [13]. As a typical natural predator of agricultural pests, the ladybird beetle, *Coccinella septempunctata* (Linnaeus), is economically important for controlling aphids, whiteflies, mites, thrips, and lepidopteran pests [6]. In Europe, Asia, and North America, this natural enemy has been commercially mass-cultured and widely employed in greenhouses and farmlands [14]. Depending on the geographical distribution of distinct populations, adults of *C. septempunctata* can enter diapause in the summer or winter [15]. In northern China, *C. septempunctata* adults enter winter diapause in response to short photoperiods and low temperatures [16]. Our previous work indicates that the specific sensitive period for diapause-inducing in the ladybird is the new adults stage (newly emerged) [16]. Moreover, our previous studies demonstrated that the first 20 days after eclosion is the pre-diapause stage of *C. septempunctata* adult at the diapause induction condition of 18 °C, L10:D14 [16,17]. Female adults of *C. septempunctata* in diapause exhibit arrested ovarian development, increased lipid accumulation and higher cold tolerance [15,16,17]. These physiological changes provide the energy required by the insect to get through the diapause period, and thus survive under hostile environmental conditions. We also found a conspicuous series of changes in diapausing females at the proteomic and transcriptomic levels. We identified several specifically expressed genes and proteins involved in lipid storage, energy metabolism, and hormonal signaling pathways [15,18]. In addition, Xiang et al. [3] revealed the diapause-related functions of several genes involved in fatty acid biosynthesis and lipid accumulation in *C. septempunctata.* However, the upstream regulators that target these genes to activate diverse physiological pathways are unclear.

An intriguing but not fully understood problem is why, according to our previous transcriptome data, the *cathepsin L* (*CsCatL*) gene is highly expressed in *C. septempunctata* during diapause. Cathepsins, a class of essential cysteine proteases, are widely distributed in animals, plants, and other organisms. Thus far, more than 20 types of cathepsins have been found, including cathepsin-A, -B, -C, -D, -E, -F, -G, -H, -K, -L, -O, -S, -V, -W, and -X, belonging to cysteine, aspartic, and serine protease families [19]. As multifunctional proteins, cathepsins play major roles in the metabolic processes of development, growth, and metamorphosis of insect. In *Antheraea pernyi*, knockdown of *cathepsin L* by RNA interference leads to larval death before pupation or abnormal phenotypes during metamorphosis [20]. The suppression of cathepsin in silkworms limits the degradation of the silk gland during metamorphosis [21]. In *Helicoverpa armigera*, cathepsin L participates in remodeling the midgut [22] and fat body degradation [23]. Furthermore, cathepsin L is regulated by 20-hydroxyecdysone (20E) [24,25] or ecdysone-transcription factors [26]. Subsequently, cathepsin L was found to be involved in the degradation of the fat body and in programmed cell death in *Bombyx mori* [19]. Although cathepsins play major roles in fat body function of insect, little is known about whether cathepsin L affects fat accumulation and other phenotypes during diapause in *C. septempunctata*.

Therefore, in the current study, we investigated the function of *CsCatL* in *C. septempunctata* during diapause. We cloned the *CsCatL* gene and used reverse transcription-quantitative polymerase chain reaction (RT-qPCR) to detect the gene expression levels during non-diapause and diapause induction. RNA interference (RNAi) technology was used for genetic silencing to investigate the functions of *CsCatL* during the diapause process. Understanding the mechanisms of the *CsCatL* gene in regulating diapause can help improve long-distance shipping and long-term storage of this insect. In addition, these results can provide a theoretical basis for a more in-depth understanding of diapause molecular regulation and suggest techniques that could be exploited for improving the shelf-life and commercial application of *C. septempunctata* as an agent of biological pest control.

## 2. Results

### 2.1. Molecular Cloning and Sequence Analysis of the CsCatL Gene

**Figure 1:**
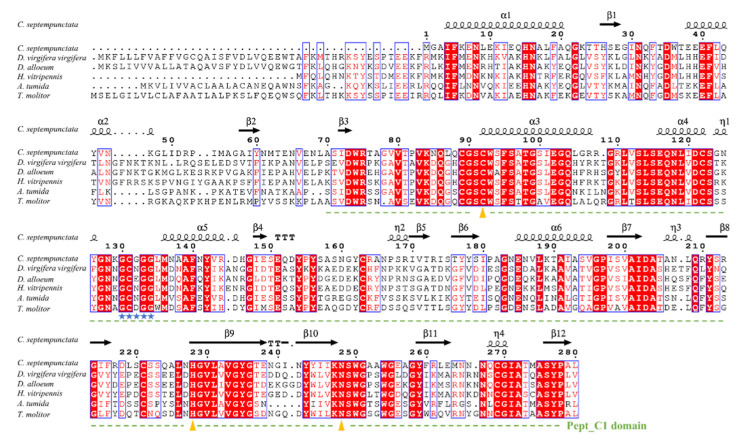
Multiple-sequence alignment of *C. septempunctata* CatL with the corresponding proteins from other insect species. Alpha-helices, eta-helices, beta strands, and beta turns are marked by α, η, β and TT, respectively. The conserved Pept_C1 domain is underlined. The active sites of Cys139-His278-Asn299 and the domain containing the “GCXGG” motif are labeled with yellow triangles and blue stars, respectively. GenBank accession numbers are: *Coccinella septempunctata* (this study), *Diabrotica virgifera virgifera* (XP_028133575.1), *Diachasma alloeum* (XP_015123049.1), *Homalodisca vitripennis* (KAG8293399.1), *Aethina tumida* (XP_019868286.1), and *Tenebrio molitor* (AAR05023.1).

**Figure 2:**
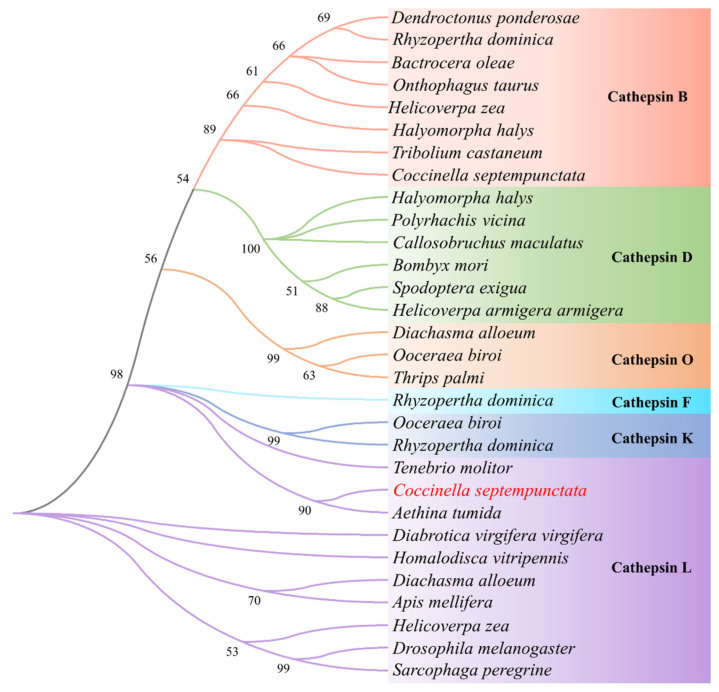
Phylogenetic tree of cathepsin proteins from *C. septempunctata* and other insects. The phylogenetic tree was constructed based on amino acid sequences of cathepsin using the WAG-based neighbor-joining method with 1000 bootstraps. *C. septempunctata* cathepsin L was highlighted in red.

Rapid amplification of cDNA-ends (RACE) was used to amplify the full-length sequences of the *CsCatL* gene from *C. septempunctata*. The cDNA sequence of *CsCatL* is 971 bp in length, containing an ORF of 843 bp. It encodes a protein of 280 amino acid residues with a predicted molecular mass of 30.55 kDa and a theoretical isoelectric point of 5.63. A conserved Pept_C1 domain as a papain-like cysteine peptidase exists between residues 70 and 278 of the deduced CsCatL protein (Figure 1). By aligning the CsCatL protein sequence with orthologs from different insect species, we found that CsCatL contains the active sites of Cys139-His278-Asn299 and a domain containing the “GCXGG” motif, which is a typical cathepsin L conserved sequence (Figure 1). A phylogenetic tree was constructed to investigate the phylogenetic relationships of the cathepsin protein. The results showed that CsCatL was assigned into cathepsin L and had a close relationship with CatL from *Aethina tumida* (Figure 2).

### 2.2. Stage-Specific Transcript Abundance of the CsCatL Gene in the Diapause Induction Phase and Non-Diapausing Phase

**Figure 3:**
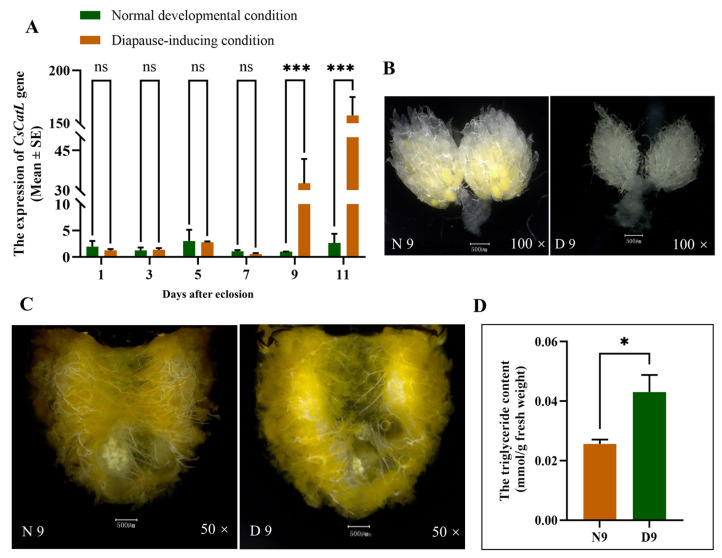
(**A**) Quantitative real-time PCR analysis of *CsCatL* relative expression during the diapause induction phase (D1, D3, D5, D7, D9, and D11) and during normal development days (N1, N3, N5, N7, N9, and N11). The mRNA expression levels of *CsActin* and *CsCatL* were measured at various time points, and data are presented as mean ± SE of triplicate biological replicates. Asterisk and “ns” respectively indicate significant difference (*** *p* < 0.001) and no significant difference (*p* > 0.05) (*t*-test); (**B**) ovarian development in N9 and D9; (**C**) lipid accumulation status; (**D**) the total triglycerides contents were measured in N9 and D9, and data are presented as mean ± SE of four biological replicates. Asterisk indicates significant difference (*t*-test; * *p* < 0.05).

We investigated the transcriptional profiles of the *CsCatL* gene at the diapause induction phase (D1, D3, D5, D7, D9, and D11) and during the corresponding developmental state (N1, N3, N5, N7, N9, and N11) of *C. septempunctata* females, respectively (Figure 3A). In the early diapause induction-phase, the mRNA abundance of the *CsCatL* gene was at the lowest level, which is close to the expression in normal development individuals. However, compared to the normal development condition, the transcript levels of the *CsCatL* significantly increased from day 9 and day 11 following diapause induction. In comparison with nondiapausing adults, the expression of *CsCatL* at D9 (*t* = 4.730, *p* < 0.001) and D11 (*t* = 23.24, *p* < 0.001) increased 30- and 100-fold, respectively.

### 2.3. Phenotypes of Females Abdomen and Ovary and Triglycerides Content in D9 and N9

To compare the status of adults in 9 days under diapause induction phase (D9) and normal development condition (N9), we investigated the phenotypes of females’ abdomen and ovary and triglycerides content in D9 and N9 in Figure 3, respectively. Compared to the normal adults, the inhibition of ovarian development (Figure 3B) and lipid accumulation were obvious (Figure 3C) with significantly increased triglycerides content (Figure 3D) in diapause adults.

### 2.4. Silencing the CsCatL Gene Reduced Lipid Accumulation in Diapause-Destined Adults of C. septempunctata

**Figure 4:**
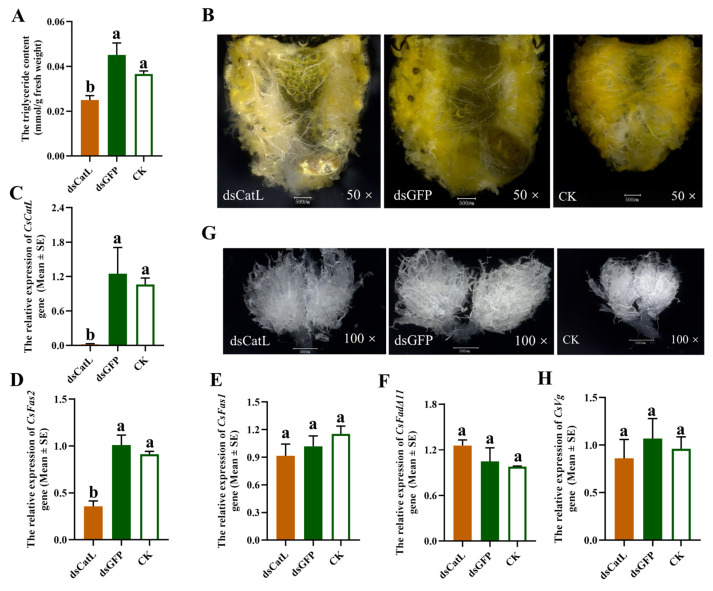
Knockdown of *CsCatL* in diapause-destined females reduces lipid accumulation. (**A**) The triglyceride content was measured in N9 and D9. Relative expressions of *CsCatL* (**C**), *CsFas1* (**E**) *CsFas2* (**D**), *CsFadΔ11* (**F**) and *CsVg* (**H**), lipid accumulation status (**B**) and ovary development (**G**) were determined on the 7th day after injection with dsRNA. Data are presented as mean ± SE (one-way ANOVA). The letters “a” and “b” respectively indicate significant difference (*p* < 0.05) and no significant difference (*p* > 0.05).

To investigate the function of the *CsCatL* gene during diapause induction phase, we used RNAi to silence the *CsCatL* gene. Injecting dsCsCatL into diapause-destined female adults significantly decreased the expression levels of *CsCatL.* Compared to the dsGFP control and CK (non-treated controls), *CsCatL* mRNA abundance was reduced by 98.4% on the 7th day post dsRNA injection (Figure 4C). In addition, knocking down the *CsCatL* significantly reduced the lipid storage. The lipid storage (Figure 4B) and triglycerides content (Figure 4A) significantly dropped. The expression of the *CsFas2* gene was reduced (Figure 4D). However, there were no significant differences in the transcriptional levels of *CsFas1* and *CsFadΔ11* (Figure 4E,F). Nevertheless, silencing *CsCatL* did not affect ovarian suppression, as evidenced by undeveloped ovaries and no significant differences in the *CsVg* mRNA abundance (Figure 4G,H). Knockdown of *CsCatL* reduced expression of genes involved in fatty acid synthesis and lipid accumulation in the diapause-destined female adult of *C. septempunctata*. There were no significant differences in the expression data, triglycerides content and phenotypic changes between dsGFP injection and a non-treated control (CK).

### 2.5. Silencing of the CsCatL Gene Did Not Affect JH Pathway Related Genes in C. septempunctata

**Figure 5:**
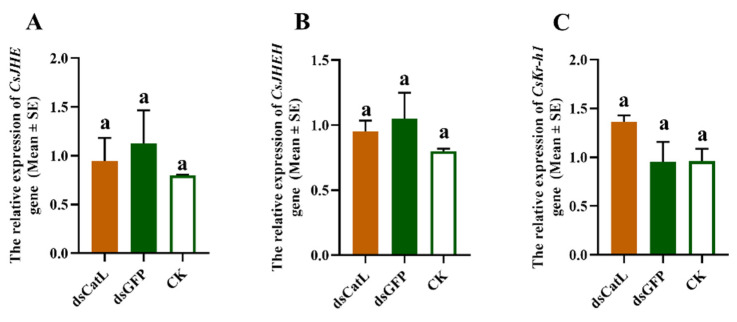
Relative expressions of *CsJHE* (**A**), *CsJHEH* (**B**), and *CsKr-h1* (**C**) were determined on the 7th day after injection of dsRNA. Data are presented as mean ± SE (one-way ANOVA). The letters “a” indicates no significant difference (*p* > 0.05).

To investigate the effect of *CsCatL* on JH pathway related genes, we tested the expression of JH esterase (*CsJHE*), JH epoxide hydrolase (*CsJHEH*) and *Krüppel-homolog* (*CsKr-h1*) after injection with dsRNA. The mRNAs abundance of *CsJHE* (Figure 5A), *CsJHEH* (Figure 5B) and *CsKr-h1* (Figure 5C) were not significantly different between the control (dsGFP and CK) and dsCsCatL treatments. Silencing *CsCatL* did not affect the mRNAs abundance of genes involved in JH pathway of *C. septempunctata*.

### 2.6. Deletion of CsCatL Resulted in a Low Survival Rate of C. septempunctata

**Figure 6:**
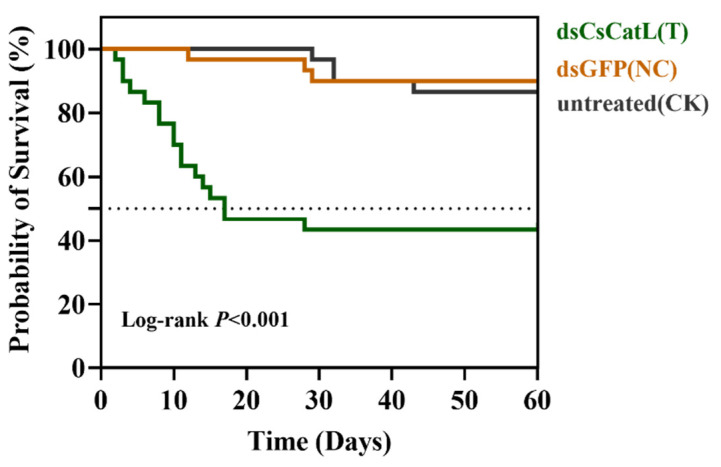
Survival of *C. septempunctata* adults after RNAi treatment. Three treatments were set up (*n* = 30): injected dsCscatL (T); injected dsGFP as a negative control (NC); untreated as a blank control (CK); the survival of adults was visually assessed daily, and the test was continued for 60 days. The data are shown as the mean ± SE. (Kaplan-Meier analysis test, *p* < 0.001).

According to the Kaplan−Meier analysis of survival data for 60 days, the survival rates of *C. septempunctata* were not significantly different between NC and CK treatments (Log-rank test: χ^2^ = 0.1362, *p* = 0.7121) (Figure 6). However, injecting dsCatL into diapause-destined females significantly decreased the survival rate of adults compared to the control groups, and less than 50% survival was observed from day 20 to day 60 after dsRNA injection (log-rank test: χ^2^ = 16.27 and *p* < 0.001 for NC; χ^2^ = 15.06, *p* < 0.001 for CK) (Figure 6).

## 3. Discussion

The ladybeetle, *C. septempunctata*, is an economically crucial natural predator of agricultural pests. It is essential to study the diapause of *C. septempunctata* for improving the shelf-life and commercial application in biological control. As multifunctional proteins, cathepsin L was considered involved in insects’ development, growth, and metamorphosis. According to our previous transcriptome data, *cathepsin L* of the *C. septempunctata* (*CsCatL*) gene was highly expressed during the diapause period. At present, there are few studies on the function of the *cathepsin L* gene in *C. septempunctata*. Therefore, it is necessary to study the function of the *CsCatL* gene duringdiapause.

Our study cloned the cathepsin L-like proteinase cDNA *CsCatL* from *C. septempunctata*. *CsCatL* encodes a 280-residue protein with a predicted molecular mass of 30.55 kDa. This protein belongs to the C1A subfamily of the C1 papain family in the CA clan of cysteine peptidases. Its precursor domain contains the “GCXGG” motif, which is a typical cathepsin L conserved sequence [27]. A phylogenetic analysis showed that CsCatL was assigned into cathepsin L. The results show that CsCatL shared high sequence similarity with CatL from other Coleoptera and was more similar to CatL from *A. tumida* (Figure 2). 

To study the function of the *cathepsin L* gene in *C. septempunctata* at the diapause stage, we carried out qRT-PCR and RNAi interference experiments, respectively. Compared to the normal developmental adults, the transcript levels of the *CsCatL* significantly increased from day 9 following diapause induction (Figure 3A). To better explicate CsCatL gene function at the same time point, the females were collected and verified by the RNAi-related genes expression on the 7th day post-dsRNA injection (on the 9th day after eclosion) after the dsRNA injection (2 days after eclosion) under diapause-inducing conditions.

Subsequently, we dissected specimens and performed microscopic investigations of tissues after RNAi injection at the pre-diapause stage. Injecting dsCsCatL into diapause-destined female adults significantly reduced lipid storage (Figure 4B) and triglycerides content (Figure 4A). Generally, diapause in insects can last months to years. Although a few insects continue to consume small quantities of food during diapause, most cease feeding [13,28]. Therefore, lipid and energy reserves accumulated during the diapause preparation stage are critical for surviving the diapause [11,13,28,29]. The biosynthesis of fatty acids (FAs) is a complex multistep reaction involving multiple enzymes, including acetyl-coenzyme A carboxylase (*Acc*), fatty acid synthase (*Fas*), elongase of very long chain fatty acid (*Elo*), fatty acid desaturase (*Fad*), fatty acyl-CoA reductase (*Far*) [30]. Unlike *Fas* genes, *Acc*, *Elo*, *Fad* and *Far* genes mainly play roles in insect reproductive capacity, epidermal function [31,32,33,34], germ cell development [35,36,37] and the synthesis of insect pheromones, epidermal alcohols and waxes [38,39,40,41,42,43]. 

However, it is generally accepted that fatty acid synthase (*Fas*) regulates lipid accumulation during insect diapause [31]. Sufficient research shows that the *Fas* gene generally plays a central role in lipid accumulation in invertebrates. For example, the *Fas* gene regulates lipogenesis by converting acetyl-CoA into palmitate, leading to the production and storage of triacylglycerols (TAG) [44]. The *Fas* gene has been found to promote lipid accumulation in the mosquito *Aedes aegypti* [31]. Moreover, upregulation of *Fas* was also observed during early diapause in the mosquito *C. pipiens.* Knockdown of *Fas1* reduced lipid accumulation and decreased the overwintering survival rate of females *C. pipiens* [45,46,47]. *Fas2* also contributes to diapause preparation in a beetle by regulating the expression of genes invovled in lipid accumulation and stress tolerance [9]. Moreover, based on previous studies in our laboratory, *Fas1*, *Fas2* and Acyl-Coa Δ11(*FadΔ11*) genes of *C. septempunctata* were significantly up-regulated and contributed to fat accumulation in its abdomen during diapause preparation stage [48]. Consequently, we just focused on the expression of *CsFas1*, *CsFas2* and *CsFadΔ11* genes after RNAi *CsCatL* gene. According to the qRT-PCR result, RNAi injection reduced the expression of the *CsFas2* gene (Figure 4D). Although the expression levels of *CsFas1* gene *and CsFadΔ11* gene did not change significantly (Figure 4E,F), lipid accumulation was still affected during the pre-diapause stage. In addition, silencing *CsCatL* did not affect ovarian suppression and *CsVg* mRNA abundance (Figure 4G,H). These suggested that silencing of the *CsCatL* gene does not affect the insects entering a state of reproductive diapause.

The absence of Juvenile Hormone (JH) is recognized as a key factor during reproductive diapause in several species, including *C. bowringi*, *Harmonia axyridis*, and *C. pipiens* [49,50,51,52]. JH levels are balanced by regulated biosynthesis and degradation in insect hemolymph. JH in the hemolymph and the tissues is mainly degraded by three enzymes, including JH esterase (*JHE*), JH epoxide hydrolase (*JHEH*) and JH diol kinase (*JHDK*) [53]. In previous study from our lab, we found that diapause in females of *C. septempunctata* was induced by a reduction in JH titers resulting from upregulation of *CsJHE* and *CsJHEH* [6]. It is generally known that Krüppel-homolog 1 (Kr-h1) is a zinc finger transcription factor promoting reproduction in adult insects through the transduction of the JH pathway [54]. Knocking down JH degradation genes (*CsJHE*, *CsJHEH*) clearly increased the expression levels of JH-inducible gene *Kr-h1* [6]. In addition, depletion of *CsJHE* and *CsJHEH* causes a marked reduction of lipid accumulation and reduced expression of *CsFas1* and *CsFas2* in the diapause-programmed females [6]. Therefore, we wondered whether the knockout of the *CsCatL* gene suppresses fat accumulation by affecting the JH pathway. However, silencing *CsCatL* does not affect the mRNAs abundance of *CsJHE*, *CsJHEH*, and *CsKr-h1* (Figure 5A–C). Therefore, the inhibition of fat accumulation by silencing *CsCatL*, presumably, has nothing to do with the JH pathway. In the future, we will conduct in-depth research on how *CsCatL* affects fat accumulation and the expression of the *CsFas2* gene.

A higher survival rate of insects was expected and crucial during diapause induction. We wanted to investigate whether reduction of lipid accumulation influenced adult survival. Subsequently, we did bioassay experiments of *C. septempunctata* injected with dsRNA. In these experiments, increased adult mortality was observed at the pre-diapause stage (Figure 6). All results indicate that the *CsCatL* gene plays a crucial role in the survival of adults during the diapause preparation stage. The *C. septempunctata* consumed small quantities of food even most ceased feeding during diapause. Compared to the non-diapause females in N9, the lipid accumulation and triglyceride content were increased in D9 (diapause preparation stage) (Figure 3C,D). Lipid and energy reserves accumulated during the diapause preparation stage were critical for the survival of the diapausing *C. septempunctata*. Although the effect of dsRNA starts to wear out with time, *CsCatL* mRNA abundance was reduced by 98.4% on the 7th day post-dsRNA injection (Figure 4C) and the triglycerides content and lipid storage significantly dropped (Figure 4A,B). Our previous studies demonstrated that the first 20 days after eclosion is the diapause preparation stage of *C. septempunctata* adults at the diapause induction condition. Therefore, the deletion of the *CsCatL* gene did cause insufficient fat accumulation during diapause preparation, which was also the main reason for the increased mortality of *C. septempunctata* at the first 20 days under the diapause induction condition. Therefore, we inferred that the reduction in lipid storage led to insufficient energy storage, which contributed to high mortality after entering diapause. 

A high efficiency of RNA interference is essential. Fortunately, our experimental object, *C. septempunctata*, belongs to Coleopterans. It is common knowledge that the RNA interference efficiency of Coleoptera insects is relatively high, even reaching 100% [55,56]. To ensure a better interference effect, we used a mixture of dsCsCatL1 and dsCsCatL2 for injection (Table 1). We have also supplemented the expression of the *CsCatL* gene between 48 and 72 h post dsRNA injection. According to our results this time, the *CsCatL* mRNA abundance decreased by 82.4% and 100% on the 48 and 72 h post-dsRNA, respectively (Figure S1). In addition, our previous studies found that several target genes’ mRNA abundance remained low within 11 days after injection [3,6]. The high efficiency of RNA interference in *C. septempunctata* is very convenient for our work.

**Figure 7:**
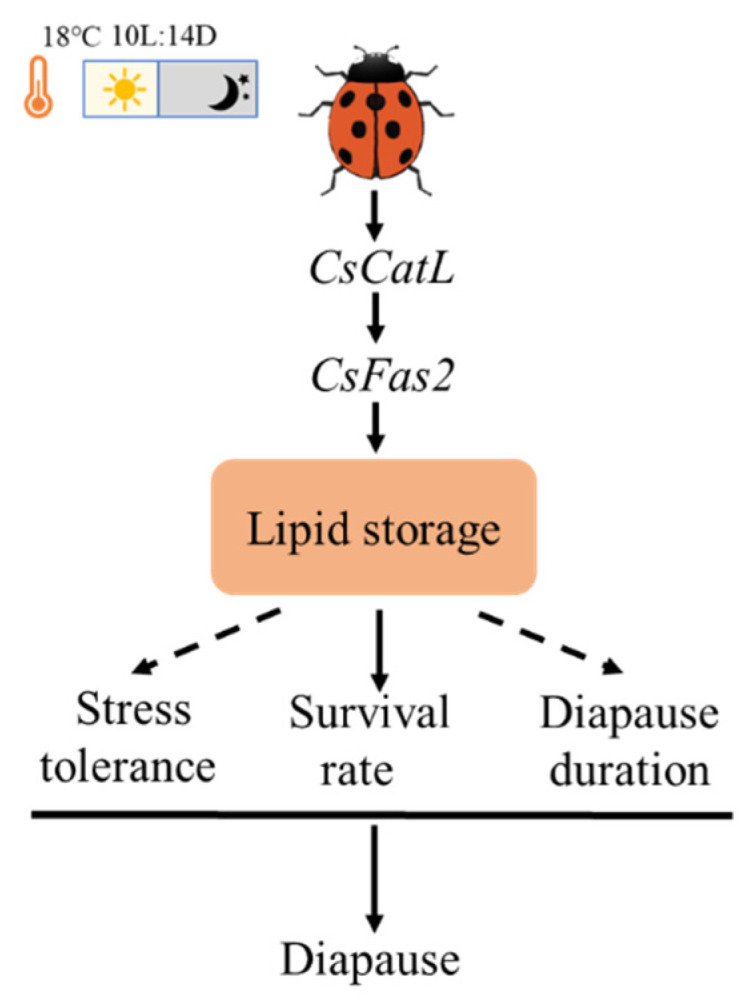
Model of how *CsCatL* promotes diapause preparation in *C. septempunctata*.

In conclusion, a novel *cathepsin L* gene was identified from *C. septempunctata*. Our results demonstrated that *CsCatL* played an essential role in fatty acid biosynthesis, lipid accumulation and survival during diapause preparation of *C. septempunctata*. We constructed a model describing how *CsCatL* promotes diapause preparation in *C. septempunctata* (Figure 7). In this model, upregulation of *CsCatL* promotes survival of diapausing *C. septempunctata* females by increasing the expression of *CsFas2* and thus significantly increases the lipid storage. This study provides a new insight into the mechanism of lipid accumulation during diapause in insects. In future research, additional physiological experiments are required to identify how accumulated lipids due to upregulation of *CsCatL* can regulate survival rates. Collectively, these studies can support the development of techniques for improving the survival rates of adults in the mass-production and commercial application of *C. septempunctata* in biological pest control.

## 4. Materials and Methods

### 4.1. Insect Rearing and Sample Preparation

A colony of *C. septempunctata* was maintained in the laboratory as described [21,24] and reared on *Aphis glycines* Matsumura at 24 ± 1 °C, with a long day photoperiod of 16L:8D, and a relative humidity (RH) of 70 ± 10% (normal developmental conditions). To induce diapause, the newly emerged adults (within 24 h after eclosion) were transferred to 18 ± 1 °C, 10L:14D and RH 70 ± 10% (diapause-inducing conditions).

To evaluate relative mRNA expression of *CsCatL* at different stages, we put newly emerged adults into 18 ± 1 °C, 10L:14D (diapause-inducing conditions) and 24 ± 1 °C, 16L:8D (non-diapause conditions/normal developmental conditions), respectively. Then, we sampled newly emerged female adults (0 day), non-diapausing 1, 3, 5, 7, 9, and 11 days-old female adults under normal developmental conditions (N1, N3, N5, N7, N9, and N11) and diapausing 1, 3, 5, 7, 9, and 11 days-old female adults under diapause-inducing conditions (D1, D3, D5, D7, D9, and D11). All insects were collected, cleaned, and frozen with liquid nitrogen. Then, the samples were stored in a refrigerator (−80 °C) until analysis. Each treatment was performed with three biological replicates, and each replicate contained four females. 

### 4.2. The Investigation of C. septempunctata Female in N9 and D9

To investigate the developmental status of adults in N9 and D9, we conducted a microscopic investigation and the determination of total triglycerides. In the microscopic investigation, each of the 10 females were observed to investigate the lipid storage and ovary developmental morphology in N9 and D9. The total body of females in N9 and D9 were used to measure triglycerides content, respectively. The total triglycerides content of each group (N9 and D9) was examined with four biological replicates, and each replicate contained ten females. 

### 4.3. Gene Cloning and Sequence Analysis

We identified one fragment encoding putative *CsCatL* (Contig14887) based on the transcriptome database previously established in our laboratory. Total RNA was extracted from individual *C. septempunctata* using the TRIzol Reagent (Invitrogen, Carlsbad, CA, USA) following the manufacturer’s instructions. The first strand of cDNA used for internal sequence amplification was synthesized using the pEASY-Blunt Simple Cloning Vector (Transgen, Beijing, China). Specific primers (Table 1) used for polymerase chain reaction (PCR) amplification were designed with DNAMAN v.6.03 software (Lynnon Biosoft, San Ramon, CA, USA) and synthesized by TSINGKE Biological Technology Company (Beijing, China). PCR was then conducted with I-5 2×High Fidelity Master Mix (TSINGKE). The target genes were subcloned into the pEASY-Blunt Simple Cloning Vector (Transgen, Beijing, China), and sequenced by TSINGKE Biological Technology Company. Next, the SMARTer^®^ RACE cDNA amplification Kit (TaKaRa Biotechnology Co., Ltd., Dalian, Liaoning, China) was used to obtain the full-length cDNAs (Table 1). PCR products of the expected size were excised from the gels, cloned, and transformed using the In-Fusion HD Cloning Kit (TaKaRa Biotechnology Co., Ltd., Dalian, Liaoning, China) and then sequenced. Finally, the 3′UTR, the 5′UTR, and the coding DNA sequence (CDS) of the *CsCatL* gene were amplified to obtain the full-length sequences.

The amino acid sequences of *CsCatL* were deduced with the ExPASy Translate tool (https://web.expasy.org/translate/ (accessed on 12 August 2022)) and physiochemical features of the proteins were predicted using ExPASy Protparam (https://web.expasy.org/protparam/ (accessed on 15 August 2022)). The conserved domain analysis was conducted using the CDD Search program of the National Center for Biotechnology Information (NCBI) web server. To investigate the evolutionary relationships of *CsCatL*, CsCatL amino acid sequences of various species (Table 2) were downloaded from the National Center for Biotechnology Information (NCBI) and aligned with sequences of *CsCatL* using ClustalW 2 and ESPript 3.0 webserver. For rendering protein sequence similarities and secondary structure information, the model of *CsCatL* protein was constructed with SWISS-MODEL (https://swissmodel.expasy.org/ (accessed on 15 August 2022)). The phylogenetic tree was constructed based on amino acid sequences of cathepsin from *C. septempunctata* and other insects (Table 2). The tree was constructed by the neighbor-joining method using the best-fit nucleotide substitution model (WAG), and a bootstrap analysis with 1000 replicates in MEGA v6.0 software.

### 4.4. Analysis of Gene Transcript Abundance by qRT-PCR

Gene transcript abundance analyses were conducted using Real-Time PCR (qRT-PCR). Two sets of experiments were conducted, including stage-specific and RNAi-related transcript abundance analyses during the diapause preparation. Stage-specific, transcript abundance analysis was performed using the timeline samples (N1, N3, N5, N7, N9, N11, D1, D3, D5, D7, D9, and D11). RNAi-related transcript abundance was examined seven days after injection.

Total RNA was extracted from the insect samples using TransZol Up (TransGen Biotech, Beijing, China). One μg of total RNA was reverse transcribed to cDNA using TransScript^®^One-Step gDNA Removal and cDNA Synthesis SuperMix (TransGen Biotech, Beijing, China) under the following conditions: 25 °C for 10 min, 42 °C for 15 min, and 85 °C for 5 s. Subsequently, real-time PCR was performed using TOROGreen^®^ 5G qPCR Premix (Toroid Technology Limited, Shanghai, China) and a LightCycler^®^ 96 Instrument (Roche (China) Holding Ltd, Shanghai, China). All reactions were run in triplicate in a total volume of 20 μL containing ten μL TOROGreen Premix, 0.8 μL of each specific primer (Table 1), 1 μL sample cDNA, and 7.4 μL nuclease-free water. PCR amplifications of the genes were conducted under the following conditions: 95 °C for 5 min, followed by 40 cycles at 98 °C for 10 s and 55–59 °C for 20 s. After the reactions were complete, CT values were determined using fixed threshold settings. The relative expression levels of genes were analyzed using the comparative 2-^ΔΔCt^ quantitation method [57] and normalized to the *CsActin* transcript level [13,16]. A total of 3 independent biological samples were included, and three technical replicates of each biological sample were processed for all reactions.

### 4.5. RNA Interference (RNAi)

To investigate the function of *CsCatL* during diapause development of *C. septempunctata*, RNA interference (RNAi) was used to suppress gene expression in female adults of *C. septempunctata*. A 303 bp and 600 bp region of *CsCatL* were amplified from cDNA by PCR with the specific primers (dsCsCatL1 and dsCsCatL2), respectively (Table 1). One μg DNA template was synthesized by the MEGAscript T7 High Yield Transcription Kit (Invitrogen, Carlsbad, CA, USA). Double-stranded RNA (dsRNA) against the green fluorescent protein (GFP) was used as the control. The dsRNA injection was performed using females of similar size and weight reared for 1 day (2 days after eclosion) under diapause-inducing conditions. Each female was injected at the internode membranes between the second and third abdominal segments with 1 μL (2 μg/μL) of dsRNA solution for each target gene. The two control groups were injected with an equivalent amount of dsRNA-GFP solution and non-treated (CK). After injection, the insects were reared in pairs under diapause-inducing conditions. The whole insects were collected seven days after injection and stored at −80 °C for subsequent RT-qPCR experiments and for microscopic investigation.

### 4.6. Quantification of the Lipid Accumulation, Gene Real-Time Quantification and Bioassay of C. septempunctata Injected with dsRNA

To evaluate whether knockdown of *CsCatL* could influence the reproductive diapause features of *C. septempunctata*, we conducted four RNAi experiments: determined the expression of related genes (*Fas*, *FadΔ11, JHE*, *JHEH*, *Kr-h1*), surivival rate, triglycerides content, visual changes in lipid storage and the ovary developmental morphology. In all RNAi experiments, females reared for 1 day (2 days after eclosion) were injected with dsRNA and collected seven days after injection. (1) In the expressions of related genes experiments, RNAi-related transcript abundances were examined seven days after injection. Each treatment was performed with three biological replicates, and each replicate contained four females. (2) In microscopic investigation after injection, a total of 10 females were observed for each treatment to investigate the visual changes in lipid storage and the ovary developmental morphology. (3) In the determination of triglyceride content experiments, triglyceride content was examined after injection. Each treatment was performed with four biological replicates, and each replicate contained ten females. (4) In the survival rate tests, the females injected with dsCsCatL, dsGFP and untreated were reared in pairs under diapause-inducing conditions (18 °C 10L:14D), respectively. Each pair of *C. septempunctata* adults were transferred to a transparent plastic cup (7 cm diameter × 9 cm height) and fed with *A. glycines* every day. A total of 30 females were observed for each treatment. The survival state of adults was recorded daily for 60 days. 

### 4.7. Microscopic Investigation

Ovarian development and the state of lipid storage were evaluated on day ten following RNAi using a Leica VHX-2000 stereomicroscope (Keyence (China) Co., Ltd, Beijing, China) and photographed digitally. For each treatment, ten females were used for measurement.

### 4.8. The Determination of Triglyceride

The Triglycerides Assay Kit (Nanjing Jiancheng, A110-1-1, GPO-PAP system, Nanjing, China) was used to measure triglycerides in order to quantify the lipid accumulation in insects after the RNAi experiment. Triglycerides content was estimated using a glycerol-3-phosphate oxidase phenol + aminophenazone (GPO-PAP) system. The prepared solutions were added to 96-well plates and compared using a microplate reader (Gene 5 CHS 3.11, BioTek, Winooski, VT, USA) under A500 nm.

### 4.9. Statistical Analysis

GraphPad Prism 9.0 (GraphPad Software, San Diego, CA, USA) was used for all statistical analyses. Student’s *t*-test was used to compare differences of the triglyceride content (in N9 and D9) and the expression of *CsCatL* at different stages. The survival rates of *C. septempunctata* under different treatments in the RNAi experiment were compared via the Kaplan-Meier analysis for abnormally distributed data. The triglyceride content and the expression of RNAi-related genes under different treatments in the RNAi experiments were analyzed with one-way ANOVA.

## Figures and Tables

**Table 1:** The primers used in this study.

Primer Name	Sequence (5′-3′)	
Gene cloning		
*CsCatL* F	GAAGACAACCCATTCCGAGG	
*CsCatL* R	CTGCAAGAAAGGTCCCTGAA	
*CsCatL* 3RACE F1	GATTACGCCAAGCTTCCTACTCGGCATCTAACGGATAC	
*CsCatL* 3RACE F2	GATTACGCCAAGCTTCAGCTATTGCTAGTGTGGGTCC	
*CsCatL* 5RACE F1	GATTACGCCAAGCTTGGTCTGTCTATCAAACCCTTGTTAAC	
*CsCatL* 5RACE F2	GATTACGCCAAGCTTGTATCCGTTAGATGCCGAGTAGG	
qRT-PCR		
*CsCatL* F	CACGGAGTCCTAGCTGTTGG	
*CsCatL* R	GGAAGCCATGGTGGCAATTC	
*CsFas1* F	CCGTAGTCTGCCAAACATCC	
*CsFas1* R	AAATCCTCAACAGCAACGACTC	
*CsFas2* F	TTTGGCGATAGAACATAGAGCA	
*CsFas2* R	AGCCCACGGACAGGAAC	
*CsFadΔ11* F	CTGCTAACTGAGGAACTTGTGG	
*CsFadΔ11* R	GCACCAACATAACCATAGGGA	
*CsVg* F	AAACACTCCAATGCGGTC	
*CsVg* R	GAGAATGATGTAGGCAGCG	
*CsActin* F	GATTCGCCATCCAGGACATCTC	
*CsActin* R	TCCTTGCTCAGCTTGTTGTAGTC	
*CsJHE* F	GACCAAAACCTTGCCCTACG	
*CsJHE* R	CCACAAACATAGAGCACTTCCG	
*CsJHEH* F	CTTTCAAAATCGCCGTTCC	
*CsJHEH* R	AAGTCCAGCACTGATTTCGTG	
*CsKr-h1* F	CAAGTGTGAGGTCTGTTCTAGGG	
*CsKr-h1* R	GGCATACTTGACAGACGTAAGG	
RNAi experiment		
dsCatL F1	TAATACGACTCACTATAGGGTGGCTGGGGCTATTTACAAC	
dsCatL R1	TAATACGACTCACTATAGGGAGGGGTAGTCCTGTTCACTC	
dsCatL F2	TAATACGACTCACTATAGGGGAAGACAACCCATTCCGAGG	
dsCatL R2	TAATACGACTCACTATAGGGCTGCAAGAAAGGTCCCTGAA	
dsGFP F	TAATACGACTCACTATAGGGCACAAGTTCAGCGTGTCCG	
dsGFP R	TAATACGACTCACTATAGGGAGTTCACCTTGATGCCGTTC	

**Table 2:** Information on the insects included in the phylogenetic analysis.

Order	Species	GenBank/Uniprot No.
*Cathepsin L*	*Coccinella septempunctata*	This study
-	*Aethina tumida*	XP_019868286.1
-	*Tenebrio molitor*	AAR05023.1
-	*Diabrotica virgifera virgifera*	XP_028133575.1
-	*Homalodisca vitripennis*	KAG8293399.1
-	*Diachasma alloeum*	XP_015123049.1
-	*Apis mellifera*	XP_625135.3
-	*Helicoverpa zea*	XP_047024875.1
-	*Drosophila melanogaster*	Q95029.2
-	*Sarcophaga peregrine*	Q26636.1
*Cathepsin B*	*Rhyzopertha dominica*	KAI7815596.1
-	*Bactrocera oleae*	XP_014086025.1
-	*Onthophagus taurus*	XP_022913411.1
-	*Halyomorpha halys*	XP_014290885.1
-	*Helicoverpa zea*	XP_047024476.1
-	*Coccinella septempunctata*	XP_044754716.1
-	*Tribolium castaneum*	XP_974298.1
-	*Dendroctonus ponderosae*	XP_019755614.1
*Cathepsin D*	*Halyomorpha halys*	XP_014291624.1
-	*Polyrhachis vicina*	AEC03508.1
-	*Callosobruchus maculatus*	ACO56332.1
-	*Bombyx mori*	NP_001037351.1
-	*Spodoptera exigua*	ARE67826.1
-	*Helicoverpa armigera armigera*	AYP72766.1
*Cathepsin O*	*Diachasma alloeum*	XP_015115164.1
-	*Ooceraea biroi*	EZA49885.1
-	*Thrips palmi*	XP_034250554.1
*Cathepsin F*	*Rhyzopertha dominica*	KAI7815244.1
*Cathepsin K*	*Ooceraea biroi*	EZA49955.1
-	*Rhyzopertha dominica*	KAI7815176.1

## Data Availability

Not applicable.

## Supplementary Materials

The following supporting information can be downloaded at: https://www.mdpi.com/article/10.3390/ijms24010611/s1.
