# Supplementary material for: Cathepsin L Contributes to Reproductive Diapause by Regulating Lipid Storage and Survival of Coccinella septempunctata (Linnaeus)
Source: Int J Mol Sci. 2022 Dec 29;24(1):611. doi: 10.3390/ijms24010611 (PMC9820742; doi:10.3390/ijms24010611)

Figure S1: Relative expressions of *CsCatL* gene after injection. (A): 48h after injection; (B): 72h after injection. Data are presented as Mean ± SE (one-way ANOVA). The letters "a" and "b" respectively indicate significant difference ($p < 0.05$) and no significant difference ($p > 0.05$).

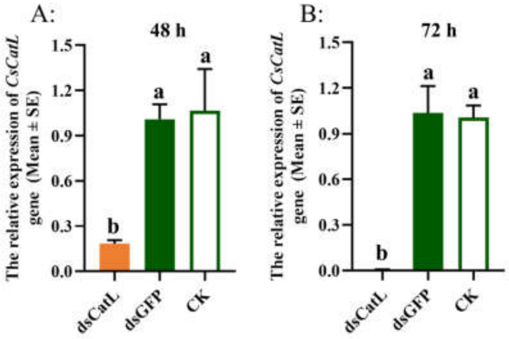

Supplement: Supplementary file 1 [file ijms-24-00611-s001.zip › Figure S1-legend.pdf]
